# Supplementary material for: Naphthalene Diimides Carrying Two β-Cyclodextrins Prefer Telomere RNA G-Quadruplex Recognition
Source: Molecules. 2022 Jun 23;27(13):4053. doi: 10.3390/molecules27134053 (PMC9268153; doi:10.3390/molecules27134053)
Supplement: Supplementary file 1 [file molecules-27-04053-s001.zip › molecules-1719858-supplementary.pdf]

Supplementary Information

Naphthalene diimides carrying two β-cyclodextrins prefer Telomere RNA G-quadruplex recognition

Tingting Zou,[1] Yuka Sato,[1] Shuma Kaneyoshi,[1] Kota Mano,[1] Rui Yasukawa,[1] Yoshifumi Nakano,[1] Satoshi Fujii,[2] Shinobu Sato,[1*] Shigeori Takenaka [1*]

[1] Department of Applied Chemistry, Kyushu Institute of Technology, Fukuoka, 804-8550, Japan
[2] Department of Bioscience and Bioinformatics Kyushu Institute of Technology, Fukuoka 820-8502, Japan
* Correspondence: shige@che.kyutech.ac.jp; Tel.: +81-93-884-3322

Tables of contents

P2: Confirmation of 1 (Figure S1)
P3: Confirmation of 1 (Figure S2), Confirmation of 4 (Figure S3)
P4: Confirmation of 4 (Figure S4)
P5: Confirmation of 2 (Figure S5)
P6: Confirmation of 2 (Figure S6)
P7: CD spectra of DNAs or RNAs with adding 2 (Figure S7)
P8: CD spectra of DNAs or RNAs with adding 3 (Figure S8)
P9: UV-Vis Spectra of 1-3 with adding DNA (Figure S9)
P10: Binding curve of 1-3 with adding DNA (Figure S10)
P11: ITC measurements of 2 or 3 with DNA or RNA (Figure S11)
P11: Binding model simulation of 2 with c-myc or 12nt of telomere RNA (Figure S12)

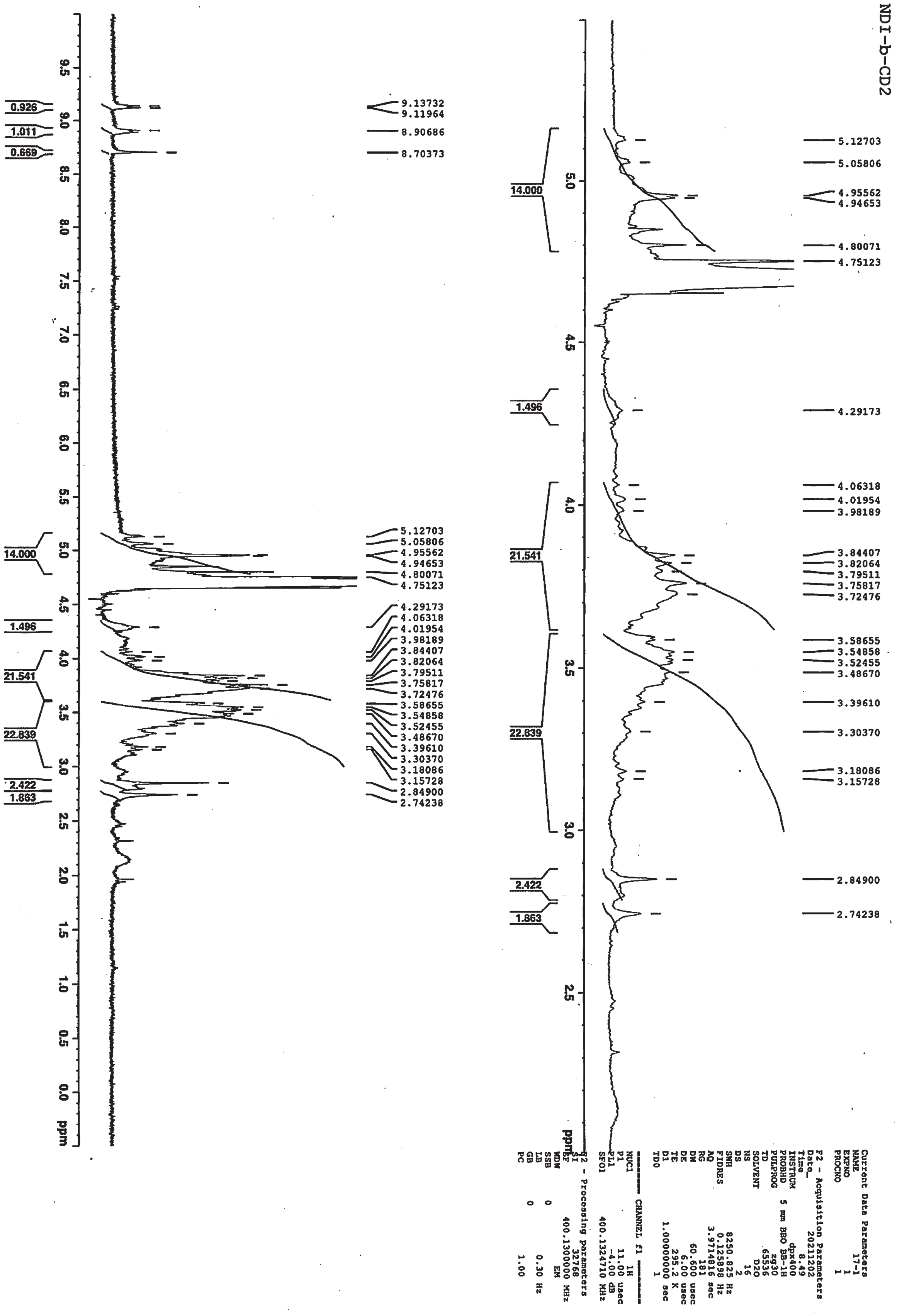

Figure S1. ^{1}H-NMR spectra of **1** in D_2O.

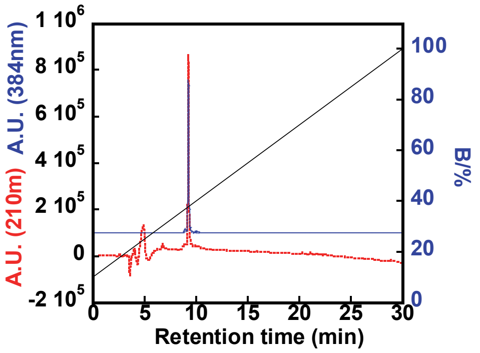

Figure S2. RT-HPLC of **1**. HPLC conditions; the concentration of solution B was changed (from 10% to 100%, 30 min) at 40 °C. Solution A: 0.1% trifluoroacetic acid, Solution B: 70% acetonitrile, 0.1% trifluoroacetic acid. Flow rate: 1 mL/min. Column: Inertsil ODS-4 (GL Sciences Inc., Tokyo, Japan).

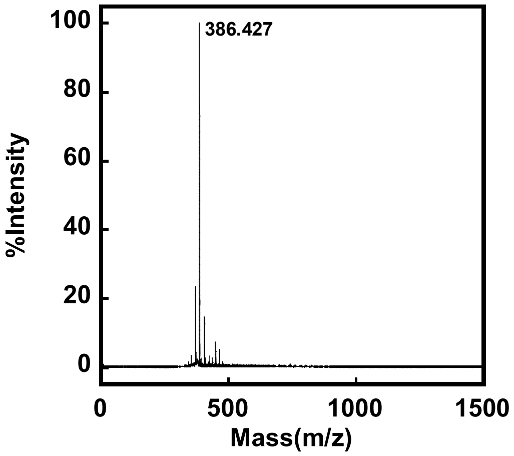

Figure S3. MALDI-TOF-MS spectra of **4** under positive mode (Matrix: DHBA). m/z = 386.427 (calculated value of $C_{18}H_{10}N_2O_8$+H^+ = 383.289).

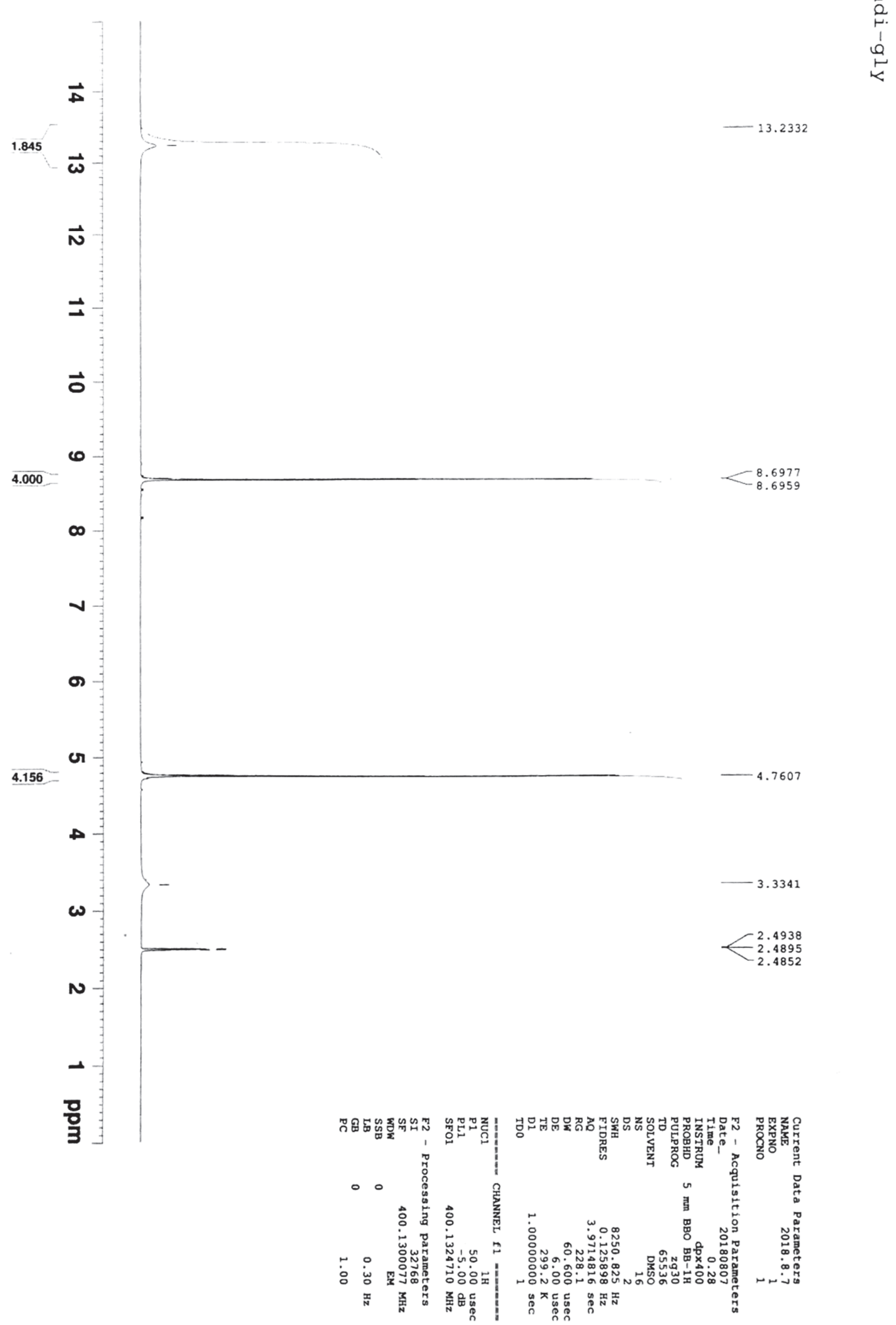

Figure S4. ^{1}H-NMR spectra of **4** in DMSO.

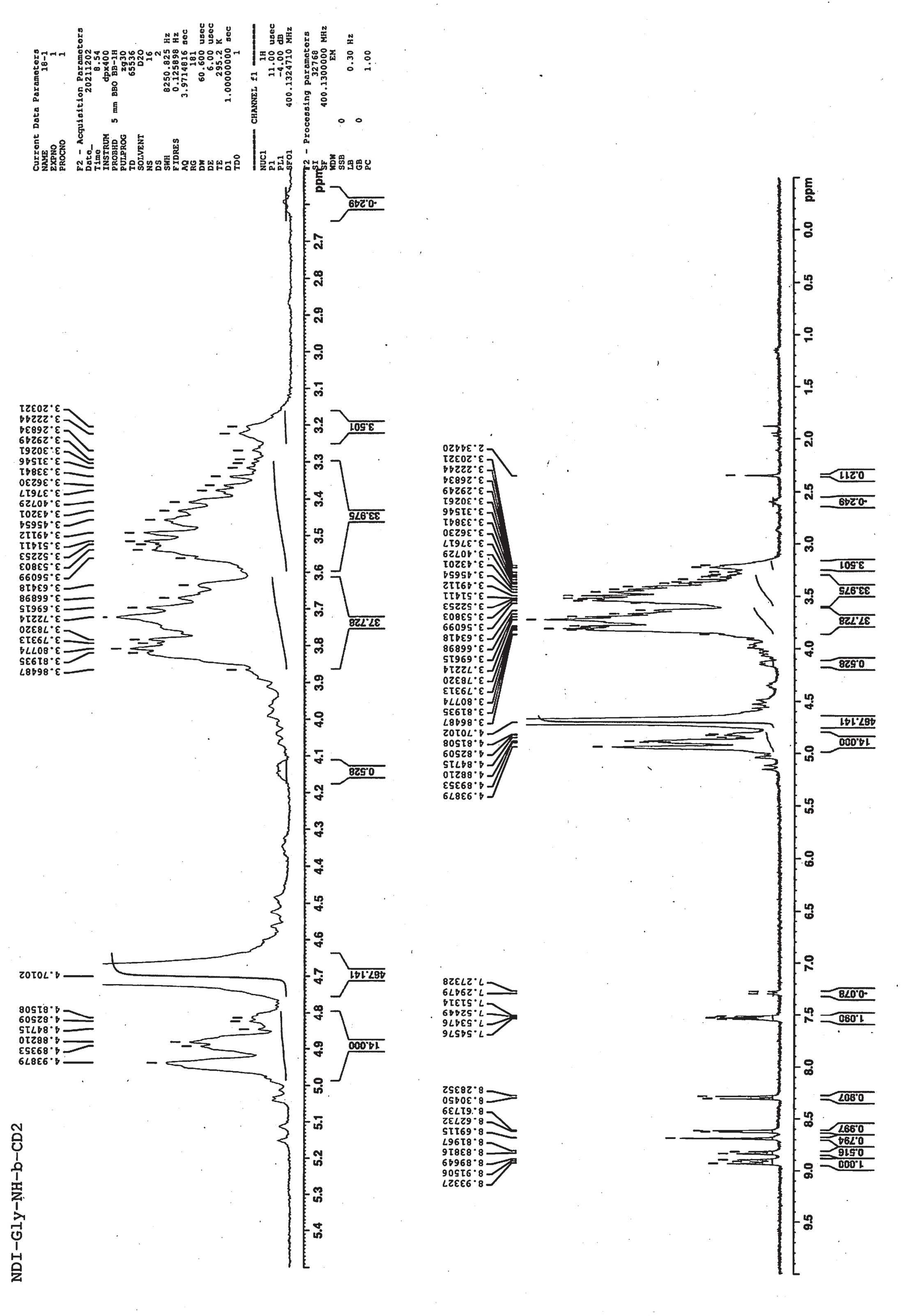

Figure S5. ^{1}H-NMR spectra of **2** in D_2O.

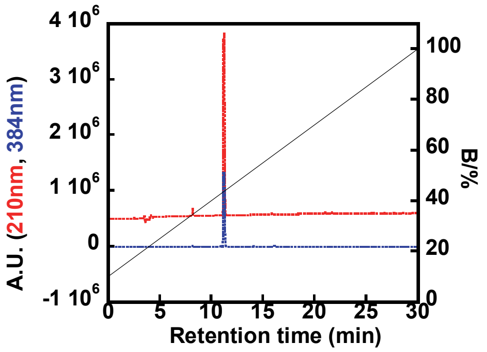

Figure S6. RT-HPLC of 2. HPLC conditions; the concentration of solution B was changed (from 10% to 100%, 30 min) at 40 °C. Solution A： 0.1% trifluoroacetic acid, Solution B：70% acetonitrile, 0.1% trifluoroacetic acid. Flow rate: 1 mL/min. Column: Inertsil ODS-4 (GL Sciences Inc., Tokyo, Japan).

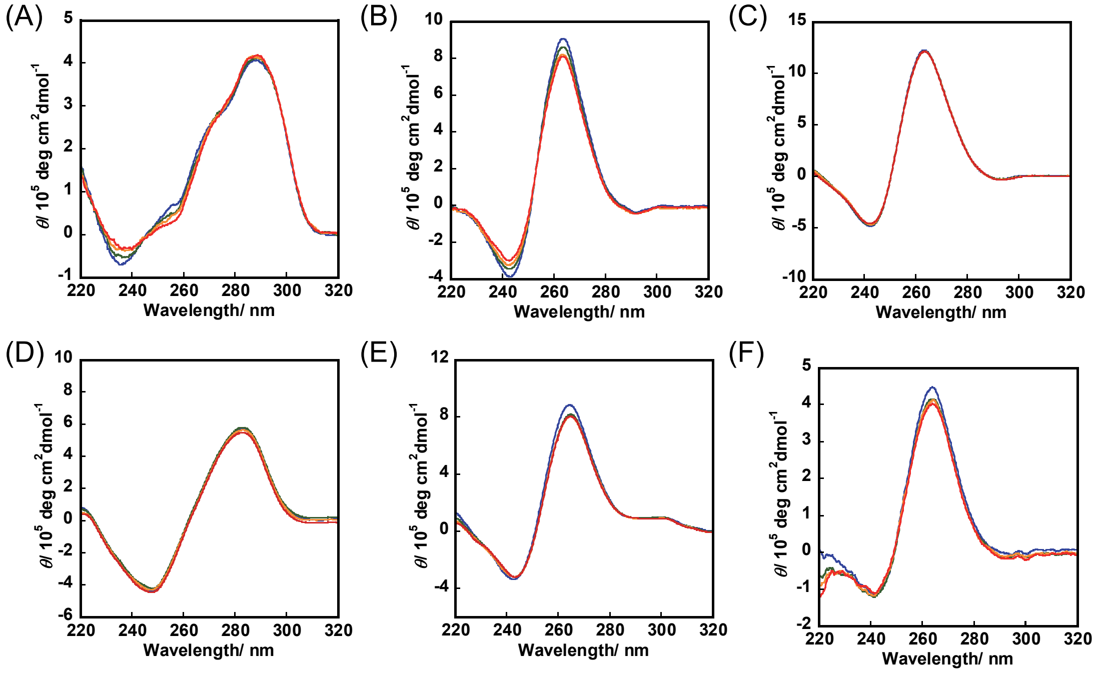

Figure S7. CD spectra of (A) Telomere G1, (B) c-myc, (C) c-kit, (D) ds-oligo, (E) G1-r23t, and (F) G1-r12t after adding **2**. 1.5 μM DNA, 0-4.5 μM **2**, 50 mM Tris-HCl buffer (pH 7.4) with 100 mM KCl at 25 °C. [DNA] : [**2**]=1:0 (blue), 1:1 (green), 1:2 (orange), 1:3 (red).

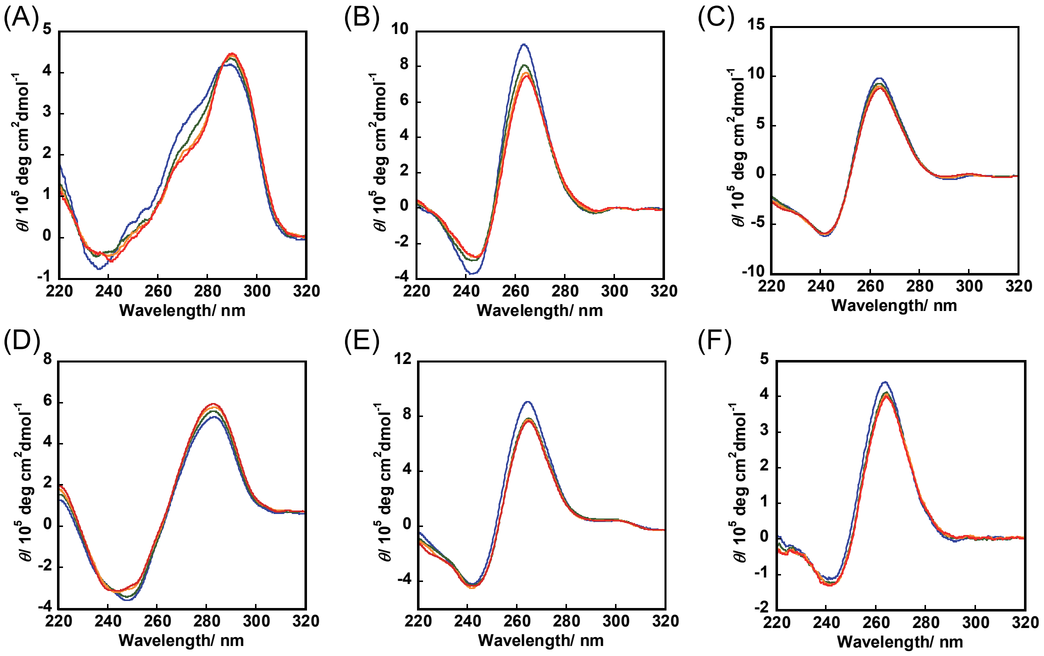

Figure S8. CD spectra of (A) Telomere G1, (B) c-myc, (C) c-kit, (D) ds-oligo, (E) G1-r23t, and (F) G1-r12t after adding **3**. 1.5 μM DNA, 0-4.5 μM **3**, 50 mM Tris-HCl buffer (pH 7.4) with 100 mM KCl at 25 °C. [DNA] : [**3**]=1:0 (blue), 1:1 (green), 1:2 (orange), 1:3 (red).

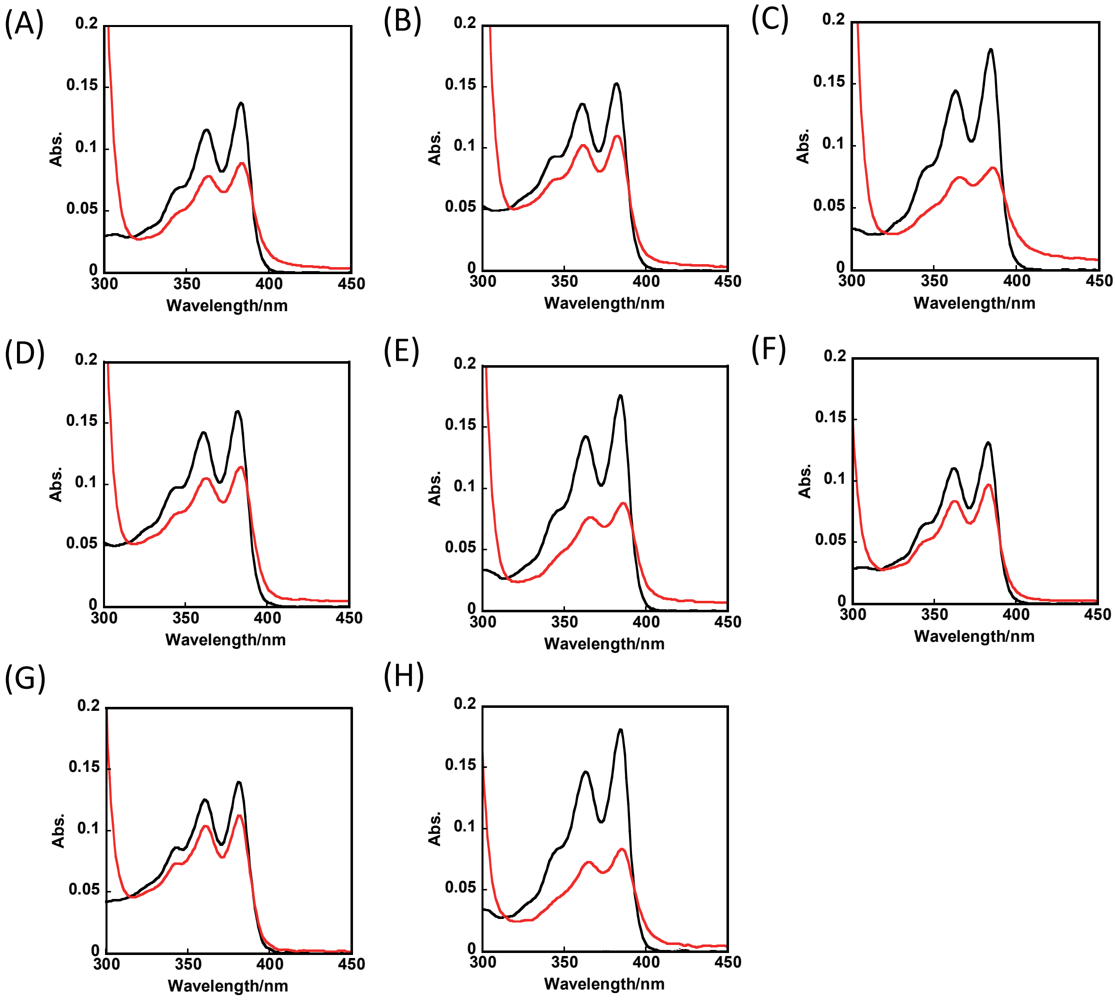

Figure S9. UV-Vis spectra of (A) **1** with Telomere G1, (B) **2** with Telomere G1, (C) **3** with Telomere G1, (D) **2** with c-myc, (E) **3** with c-myc, (F) **1** with ds-oligo, (G) **2** with ds-oligo, and (H) **3** with ds-oligo in 50 mM Tris-HCl buffer (pH 7.4) with 100 mM KCl at 25 °C (Before adding DNA: black line, after adding DNA: red line).

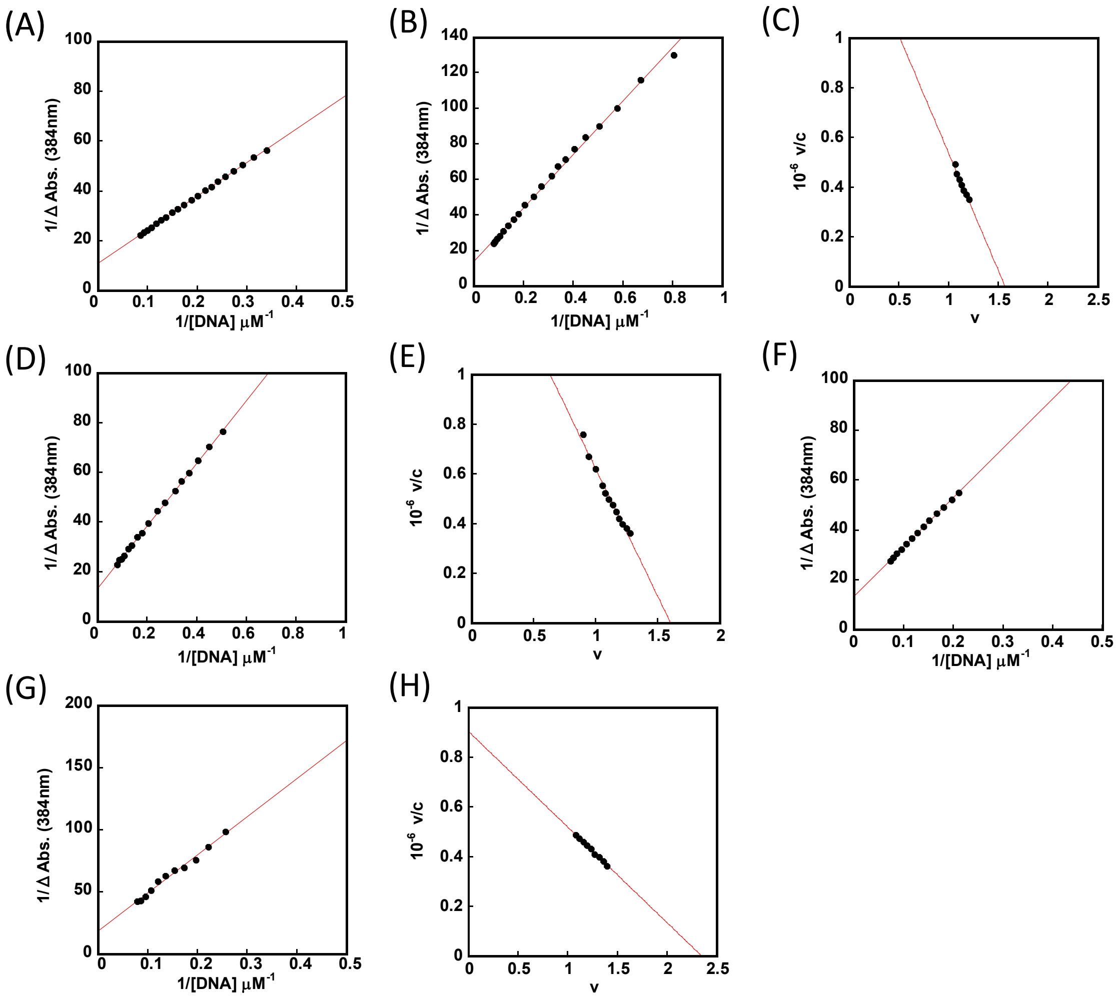

Figure S10. Binding analysis of (A) **1** with Telomere G1 (Benesi-Hildebrand analysis), (B) **2** with Telomere G1 (Benesi-Hildebrand analysis), (C) **3** with Telomere G1 (Scatchard analysis), (D) **2** with c-myc (Benesi-Hildebrand analysis), (E) **3** with c-myc (Scatchard analysis), (F) **1** with ds-oligo (Benesi-Hildebrand analysis), (G) **2** with ds-oligo (Benesi-Hildebrand analysis), and (H) **3** with ds-oligo (Scatchard analysis) in 50 mM Tris-HCl buffer (pH 7.4) with 100 mM KCl at 25 °C.

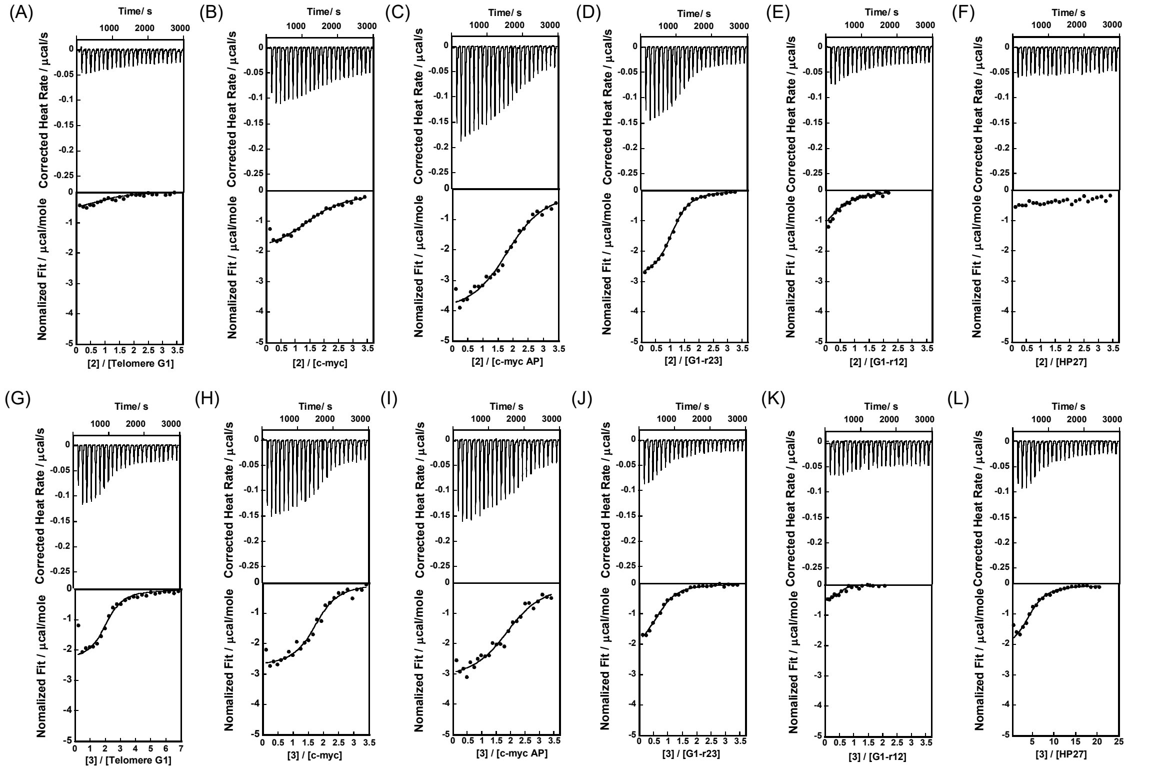

Figure S11. ITC measurement of (A, G) Telomere G1, (B, H) c-myc, (C, I) c-myc AP site, (D, J) HP27, (E, K) G1-r23nt and (F, L) HP27 with adding **2** (A-F) or **3** (G-L) in 50 mM KH_2PO_4-K_2HPO_4 buffer (pH 7.0), 25 °C.

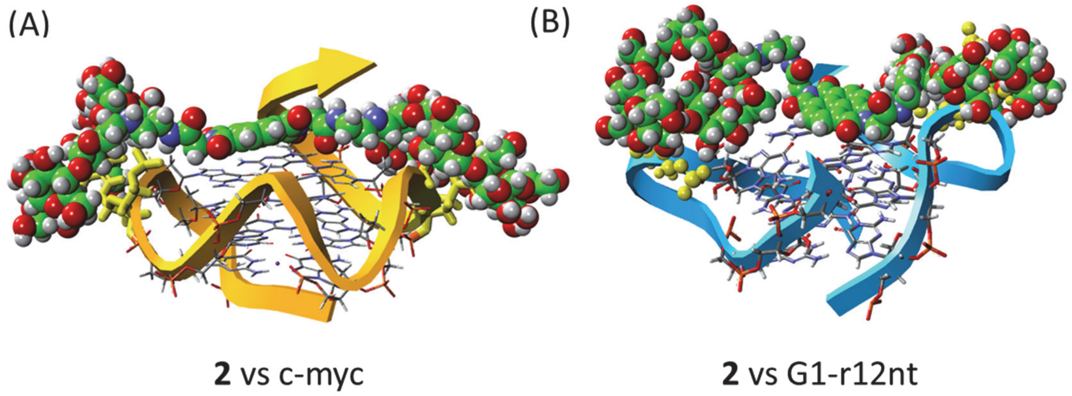

Figure S12. Binding model simulation of NDI-β-CyDs conjugator **2** with (A) c-*myc* G-quadruplex (PDB:1xav); (B) 12nt of telomere RNA G-quadruplex (PDB: 2kbp).
